# Softness Induced Enhancement in Net Throughput of Non-Linear Bio-Fluids in Nanofluidic Channel under EDL Phenomenon

**DOI:** 10.1038/s41598-018-26056-6

**Published:** 2018-05-18

**Authors:** Harshad Sanjay Gaikwad, Pranab Kumar Mondal, Somchai Wongwises

**Affiliations:** 1 Department of Mechanical Engineering, Indian Institute of Technology Guwahati, Guwahati, Assam 781039 India; 2 Fluid Mechanics, Thermal Engineering and Multiphase Flow Research Lab (FUTURE), Department of Mechanical Engineering, Faculty of Engineering, King Mongkut’s University of Technology Thonburi, Bangmod, Bangkok, 10140 Thailand; 3 Present Address: The Academy of Science, The Royal Institute of Thailand, Sanam Suea Pa, Dusit, Bangkok, 10300 Thailand

## Abstract

In this article, we describe the electro-hydrodynamics of non-Newtonian fluid in narrow fluidic channel with solvent permeable and ion-penetrable polyelectrolyte layer (PEL) grafted on channel surface with an interaction of non-overlapping electric double layer (EDL) phenomenon. In this analysis, we integrate power-law model in the momentum equation for describing the non-Newtonian rheology. The complex interplay between the non-Newtonian rheology and interfacial electrochemistry in presence of PEL on the walls leads to non-intuitive variations in the underlying flow dynamics in the channels. As such, we bring out the variations in flow dynamics and their implications on the net throughput in the channel in terms of different parameters like power-law index (*n*), drag parameter (*α*), PEL thickness (*d*) and Debye length ratio (*κ*/*κ*_*PEL*_) are discussed. We show, in this analysis, a relative enhancement in the net throughput through a soft nanofluidic channel for both the shear-thinning and shear-thickening fluids, attributed to the stronger electrical body forces stemming from ionic interactions between polyelectrolyte layer and electrolyte layer. Also, we illustrate that higher apparent viscosity inherent with the class of shear-thickening fluid weakens the softness induced enhancement in the volumetric flow rate for the shear-thickening fluids, since the viscous drag offered to the f low f ield becomes higher for the transport of shear-thickening fluid.

## Introduction

The electrostatic interaction of the charged wall with electrolytic solution under thermodynamic equilibrium leads to the formation of Electric Double Layer (EDL)^1–9^. Such phenomena upon interaction with interlaced electrical and viscous properties of the solvent permeable and ion penetrable polyelectrolyte layer grafted on the walls of narrow fluidic channel finds a wide variety of applications like an ion rectification, chemical sensing, flow control, developing devices for energy applications, characterization of gels and elastomers, manipulation and switching of ion transports to name a few^10^. This sandwiched polyelectrolyte layer between the channel walls and electrolyte solution alters the underlying flow dynamics non intuitively, as modulated by several parameters like PEL thickness, charge density and steric interactions^11,12^. The presence of such polyelectrolyte layers on the walls of the narrow fluidic channels can also be treated as the flow suppressors, since the friction drag offered by the polymeric distributions of PEL attenuates the flow rate in the channel^13^. Due to this effectiveness of polyelectrolyte layer in the flow of electrolytic solution, the PEL grafted narrow fluidic channels are called as the Soft or Smart narrow fluidic channels and the effectiveness is termed as softness of PEL. It is worth mentioning here that the softness of PEL can be varied by changing the drag parameter, PEL thickness and the concentration of electrolyte solution. Also, soft narrow fluidic channels are majorly employed in different biological and chemical applications such as electro-kinetics of biological cells, the effect of EDL in bacterial adhesion to surfaces and charging and swelling of cellulose films^14–17^.

It is important to mention here that all the aforementioned applications are largely involved with the transport of non-Newtonian bio-fluids. As such, in drug delivery applications, the emulsions comprising of the ionized oil and aqueous solution of anticancer drug follow a rheological behavior. Both the oil-water (O-W) and water-oil (W-O) emulsions show a shear-thinning nature, while W-O has higher apparent viscosity than the O-W emulsion^18^. Also, an increment in the solid particle or aggregate concentration in the pharmaceutical formulations results in an entranced reaction of solid content of suspensions to the external forcings. In fact, this phenomenon leads to an increment in the dilatancy or shear-thickening nature of the pharmaceutical formulations^19^. Considering all above pertinent issues involved with different bio-medical/biochemical applications, a thorough understanding of the underlying transport of non-Newtonian fluids through a soft narrow fluidic channel appears to be an important aspect for the modifications in the micro-electro-mechanical circuitry, primarily attributed to the complex interplay among different forcings associated with the underlying transport process. Accounting this aspect, a few studies as reported in the literature have delineated the physico-chemical interaction of electromechanics at small scales and fluid dynamics such as charge regulated surface, ion-partitioning effect, higher surface charge (relaxation effect) and surface potential, pH dependent charge density, Field Effect Transistor (FET) regulated surface potential, diffusion effect of ions, specific ion interaction, grafting of end charged polyelectrolyte brushes and their influential impacts on streaming current, streaming potential as well as electro-viscous effect^10,13,20–26^. But the non-intuitive molecular interactions of the PEL with the apparent viscosity of the non-Newtonian fluid fetches a new framework for fluid flow analysis owing to fit in the shear dependent nature of the fluid viscosity in different applications mentioned above^27^. Albeit, attention has been paid by the researchers on the flow dynamics of a Jeffrey fluid, which belongs to the category of non-Newtonian fluid as well, through a soft narrow fluidic channel^28^, the rheology driven modifications in viscous force under electro-kinetic influences and its interaction with the soft substrate together with a few pertinent aspects like the consideration of the finite thickness of PEL and the interaction of EDL formed near the walls of square/rectangular channels may bring in new flow physics, which are remaining still untouched in the literature till date.

Here, we investigate the electrohydrodynamics of non-Newtonian fluid in soft narrow fluidic channels, actuated by the combined influences of applied pressure gradient as well as applied electric field across the channel under EDL phenomenon. Due to a comparatively higher thickness of EDL in soft narrow fluidic channels than that of the rigid channels, we here assume a channel with square cross section essentially to consider the interactive effects of the EDLs being formed at two perpendicular walls of the channel on the underlying flow dynamics, which is yet to be considered in the literature. Also, in tune with what has been considered by Li *et al*.^28^, we invoke to the continuum model in this analysis for analyzing the underlying electrohydrodynamics of non-Newtonian fluids in square cross section narrow fluidic channels. We subsequently discuss the problem formulation, mathematical modeling, model validation and results of this analysis systematically.

## Mathematical Modelling

**Figure 1:**
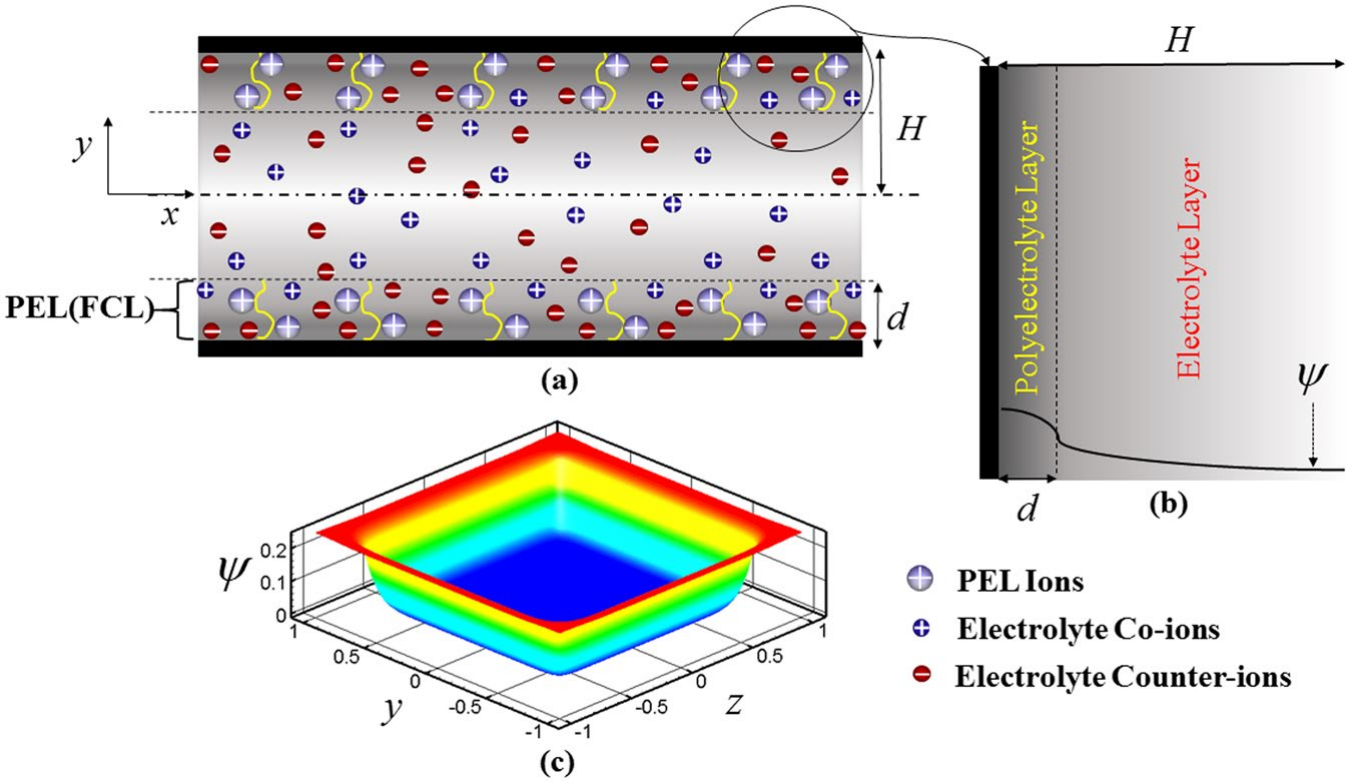
(**a**) Schematic depiction of a narrow fluidic channel with polyelectrolyte layer of thickness *d*, grafted on its walls. The height of the channel is 2*H*. The PEL is assumed to be positively charged whereas the positively charged ions of electrolyte are co-ions and negatively charged ions of electrolyte are counter-ions as shown in the schematic. (**b**) The zoomed in view of two different layers (PEL and electrolyte layer) are shown, where the variation of *ψ* is also depicted and (**c**) Figure shows the potential distribution inside the narrow fluidic channel.

In this analysis, we consider a 2D square cross section (YZ-plane) of a narrow fluidic channel whose walls are grafted with polyelectrolyte layer of thickness (*d*) as schematically depicted in Fig. 1. Note that *H* is the half height of channel, while *W* is the width of the channel. Since we here assume that the channel length is much larger than its height and width, the thermo-physical properties remain invariant with the length of the channel. We consider, in this analysis, a fully developed laminar incompressible steady flow of non-Newtonian fluid in the axial direction of the channel.

### Electrostatics

In thermodynamic equilibrium, when an electrolyte comes in contact with the charged surface, the physico-chemical interaction between the electrolyte ions and the charged surface forms the Electrical Double layer (EDL)^2,29^. On application of external electric field across the channel, which upon interaction with the EDLs formed at the channel walls, an electroosmotic flow takes place. In this study, we have considered both the driving sources *viz*. applied pressure gradient and applied electric field across the channel to make the flow occur. It should be mentioned here that the relative permittivity of the electrolyte is assumed to remain unaltered in presence of PEL^30,31^. On this basis, the potential distribution in the channel cross section according to Poisson equation can be written as^32^:

For electrolyte layer:

1 $${\varepsilon }_{r}{\varepsilon }_{0}(\frac{{\partial }^{2}{\psi }_{e}}{\partial {y}^{2}}+\frac{{\partial }^{2}{\psi }_{e}}{\partial {z}^{2}})=-\,e{z}_{e}({n}_{+}-{n}_{-})$$

For PEL:

2 $${\varepsilon }_{r}{\varepsilon }_{0}(\frac{{\partial }^{2}{\psi }_{p}}{\partial {y}^{2}}+\frac{{\partial }^{2}{\psi }_{p}}{\partial {z}^{2}})=-\,(e{z}_{e}({n}_{+}-{n}_{-})+ZeN)$$

In equations (1) and (2), *ε*_*r*_ is relative permittivity, *ε*_0_ permittivity of free space, *ψ*_*e*_ and *ψ*_*p*_ are the induced electric potentials for electrolyte and PEL respectively, *e* is the protonic charge, *z*_*e*_ and *Z* are the valency of ions in electrolyte and PEL respectively, *n*_±_ is concentration of ions in electrolyte and *N* is the concentration of PEL ions^32,33^. According to Gouy-Chapman model and Boltzmann charge distribution theory^1,27^, the charge distribution for a *z*_*e*_:*z*_*e*_ symmetric electrolyte $({\rm{NaCl}}={{\rm{Na}}}^{+}+{{\rm{Cl}}}^{-}:{\rm{KCl}}={{\rm{K}}}^{+}+{{\rm{Cl}}}^{-})$ together with the consideration of ions as point charges, is given by: ${n}_{+}={n}_{0}\,\exp (\,-\,{z}_{e}e\psi /{k}_{B}T)$; ${n}_{-}={n}_{0}\,\exp ({z}_{e}e\psi /{k}_{B}T)$, where *n*_0_ is neutral charge concentration, *k*_*B*_ is the Boltzmann constant and *T* is the absolute temperature. It should be mentioned here that the potential at the walls of the channel is assumed to be less than |25 mV|, leading to the validity of applicability of Debye-Huckel approximation $(\sinh (\frac{{z}_{e}e\psi }{{k}_{B}T})\approx \frac{{z}_{e}e\psi }{{k}_{B}T})$ in the present analysis^1^. Employing this charge distribution along with the consideration of the Debye-Huckel approximation, equations (1) and (2) can be modified as:

For electrolyte layer:

3 $$\frac{{\partial }^{2}{\psi }_{e}}{\partial {y}^{2}}+\frac{{\partial }^{2}{\psi }_{e}}{\partial {z}^{2}}={\kappa }^{2}{\psi }_{e}$$

For PEL:

4 $$\frac{{\partial }^{2}{\psi }_{p}}{\partial {y}^{2}}+\frac{{\partial }^{2}{\psi }_{p}}{\partial {z}^{2}}={\kappa }^{2}{\psi }_{p}-{\kappa }_{p}^{2}$$

In equations (3) and (4), $\kappa =\sqrt{2{{z}_{e}}^{2}{e}^{2}{n}_{0}/{\varepsilon }_{r}{\varepsilon }_{0}{k}_{B}T}$ and ${\kappa }_{p}=\sqrt{{z}_{e}Z{e}^{2}N/{\varepsilon }_{r}{\varepsilon }_{0}{k}_{B}T}$ are the Debye-Huckel parameters for electrolyte layer and the PEL respectively^32^.

### Electrohydrodynamics

To study the fluid flow through the PEL grafted narrow fluidic channel, we here employ a continuum dynamic approach^34^. The governing equations, following the continuum dynamic approach, consider that the fluid properties of the system are independent of the system parameters such as state variables, time and position. Since these state variables are dependent on the intermolecular forces in the system, the characteristic length scale and the time scale of the system depend on the intermolecular forces in the confined region near to the surface of the channels. For these scales, the energy barriers are generally set by multiple interactions such as steric interactions within the scale of ${l}_{ref,SI}$ ~ 1–2 nm, electrostatic interactions of *l*_*ref*,*EI*_ ~ 1–100 nm and van der Waals interactions of *l*_*ref*,*VI*_ ~ 1–50 nm^34^. Since we here do not consider the steric interactions, consideration of the continuum dynamic approach as employed in this analysis becomes justified for the characteristic length scale which is higher than at least 5 nm^34,35^. Accounting this, we invoke to the Cauchy momentum equation, which for the present analysis can be written as^1^:

5 $$0=-\frac{\partial p}{\partial {x}_{i}}+\frac{\partial {\tau }_{ij}}{d{x}_{j}}+{F}_{V}$$

In equation (5), *p* is applied pressure, *τ*_*ij*_ is the stress tensor and *F*_*V*_ is the volumetric body force. According to Maxwell’s stress tensor under applied electric field^36^, the electroosmotic body force is given as^32^:

For electrolyte layer:

6 $${F}_{V}=e{z}_{e}({n}_{+}-{n}_{-}){E}_{x}$$

For PEL:

7 $${F}_{V}=e{z}_{e}({n}_{+}-{n}_{-}){E}_{x}-{\mu }_{c}{u}^{n}$$

In equations (6) and (7), *E*_*x*_ is applied electric field, *μ*_*c*_*u*^*n*^ is the Darcy friction drag and *μ*_*c*_ is the Darcy friction factor. As mentioned before, the rheology of the non-Newtonian fluid is represented in this analysis by the Ostwald de’Waele Power law model. Below we write the constitutive behavior for the power-law model^37–40^:

8 $$\tau =m{(\dot{\gamma })}^{n}$$

Here, *m* is fluid consistency coefficient, *n* is power law index and $\dot{\gamma }$ is the magnitude of strain rate tensor as given by, $\dot{\gamma }=\frac{1}{2}{[{e}_{ij}:{e}_{ij}]}^{1/2}$ where *e*_*ij*_ is strain rate tensor: ${e}_{ij}=\frac{1}{2}(\frac{\partial {u}_{i}}{\partial {x}_{j}}+\frac{\partial {u}_{j}}{\partial {x}_{i}})$^41–44^. Employing this relation and using equation (8), the apparent viscosity $({\eta }_{eff}=m{(\dot{\gamma })}^{n-1})$ for two-dimensional YZ-cross section is derived as:

9 $${\eta }_{app}=m{[{(\frac{\partial u}{\partial y})}^{2}+{(\frac{\partial u}{\partial z})}^{2}]}^{(n-1)/2}$$

Using this expression equation (9), the stress tensor in x direction for both y and z planes are reduced as:

$${\tau }_{xy}=m{[{(\frac{\partial u}{\partial y})}^{2}+{(\frac{\partial u}{\partial z})}^{2}]}^{(n-1)/2}\frac{\partial u}{\partial y}\,{\rm{and}}\,{\tau }_{xz}=m{[{(\frac{\partial u}{\partial y})}^{2}+{(\frac{\partial u}{\partial z})}^{2}]}^{(n-1)/2}\frac{\partial u}{\partial z}$$

So momentum equation [equation (5)] takes the following form as given below:

For electrolyte layer:

10 $$\begin{array}{rcl}0 & = & -\frac{dp}{dx}+\frac{\partial }{\partial y}[m{({(\frac{\partial {u}_{e}}{\partial y})}^{2}+{(\frac{\partial {u}_{e}}{\partial z})}^{2})}^{(n-1)/2}]\frac{\partial {u}_{e}}{\partial y}\\  &  & +\,\frac{\partial }{\partial z}[m{({(\frac{\partial {u}_{e}}{\partial y})}^{2}+{(\frac{\partial {u}_{e}}{\partial z})}^{2})}^{(n-1)/2}]\frac{\partial {u}_{e}}{\partial z}-{\varepsilon }_{r}{\varepsilon }_{0}{\kappa }^{2}{\psi }_{e}{E}_{x}\end{array}$$

For PEL:

11 $$\begin{array}{rcl}0 & = & -\frac{dp}{dx}+\frac{\partial }{\partial y}[m{({(\frac{\partial {u}_{p}}{\partial y})}^{2}+{(\frac{\partial {u}_{p}}{\partial z})}^{2})}^{(n-1)/2}]\frac{\partial {u}_{p}}{\partial y}\\  &  & +\,\frac{\partial }{\partial z}[m{({(\frac{\partial {u}_{p}}{\partial y})}^{2}+{(\frac{\partial {u}_{p}}{\partial z})}^{2})}^{(n-1)/2}]\frac{\partial {u}_{p}}{\partial z}-{\varepsilon }_{r}{\varepsilon }_{0}{\kappa }^{2}{\psi }_{p}{E}_{x}-{\mu }_{c}{u}_{p}^{n}\end{array}$$

Here, in equations (10) and (11), *u*_*e*_ and *u*_*p*_ are the fluid velocities in electrolyte domain and polyelectrolyte layer (PEL) respectively.

#### Non-dimensionalisation of the transport equations

Here, we use the following parameters to make the transport equations dimensionless as: *l*_*ref*_  = *H*; *ψ*_*ref*_  = *k*_*B*_*T*/*ze*, ${u}_{ref}={U}_{HS}=n{\kappa }^{\frac{1-n}{n}}{(\frac{-{\varepsilon }_{r}{\varepsilon }_{0}{k}_{B}T{E}_{x}}{mze})}^{1/n}$ where *U*_*HS*_ is a Helmholtz-Smoluchowski velocity^40,45–47^. The transport equations in their dimensionless form read as:

Potential distribution:

For electrolyte layer:

12 $$\frac{{\partial }^{2}{\bar{\psi }}_{e}}{\partial {\bar{y}}^{2}}+\frac{{\partial }^{2}{\bar{\psi }}_{e}}{\partial {\bar{z}}^{2}}={\bar{\kappa }}^{2}{\bar{\psi }}_{e}$$

For PEL:

13 $$\frac{{\partial }^{2}{\bar{\psi }}_{p}}{\partial {\bar{y}}^{2}}+\frac{{\partial }^{2}{\bar{\psi }}_{p}}{\partial {\bar{z}}^{2}}={\bar{\kappa }}^{2}{\bar{\psi }}_{p}-{\bar{\kappa }}_{p}^{2}$$

Momentum equation:

For electrolyte layer:

14 $$\begin{array}{rcl}0 & = & -2{\rm{\Gamma }}+\frac{\partial }{\partial \bar{y}}[{({(\frac{\partial {\bar{u}}_{e}}{\partial \bar{y}})}^{2}+{(\frac{\partial {\bar{u}}_{e}}{\partial \bar{z}})}^{2})}^{(n-1)/2}]\frac{\partial {\bar{u}}_{e}}{\partial \bar{y}}\\  &  & +\,\frac{\partial }{\partial \bar{z}}[{({(\frac{\partial {\bar{u}}_{e}}{\partial \bar{y}})}^{2}+{(\frac{\partial {\bar{u}}_{e}}{\partial \bar{z}})}^{2})}^{(n-1)/2}]\frac{\partial {\bar{u}}_{e}}{\partial \bar{z}}+\frac{{\bar{\kappa }}^{n+1}{\bar{\psi }}_{e}}{{n}^{n}}\end{array}$$

For PEL:

15 $$\begin{array}{rcl}0 & = & -2{\rm{\Gamma }}+\frac{\partial }{\partial \bar{y}}[{({(\frac{\partial {\bar{u}}_{p}}{\partial \bar{y}})}^{2}+{(\frac{\partial {\bar{u}}_{p}}{\partial \bar{z}})}^{2})}^{(n-1)/2}]\frac{\partial {\bar{u}}_{p}}{\partial \bar{y}}\\  &  & +\,\frac{\partial }{\partial \bar{z}}[{({(\frac{\partial {\bar{u}}_{p}}{\partial \bar{y}})}^{2}+{(\frac{\partial {\bar{u}}_{p}}{\partial \bar{z}})}^{2})}^{(n-1)/2}]\frac{\partial {\bar{u}}_{p}}{\partial \bar{z}}+\frac{{\bar{\kappa }}^{n+1}{\bar{\psi }}_{p}}{{n}^{n}}-{\alpha }^{2}{\bar{u}}_{p}^{n}\end{array}$$

In equations (12–15), we obtain a few dimensionless parameters, which are influencing the flow dynamics through the channel. Below we write these parameters as:

$${\bar{u}}_{e},{\bar{u}}_{p}={u}_{e},{u}_{p}/{U}_{HS};\,\bar{y},\bar{z}=y,z/H;\,\bar{\kappa }=\kappa H;\,{\bar{\psi }}_{e},{\bar{\psi }}_{p}={\psi }_{e},{\psi }_{p}/{\psi }_{ref}$$

$${\rm{\Gamma }}=\frac{1}{2}\frac{dp}{dx}/(\frac{m{U}_{HS}^{n}}{{H}^{n+1}}):\,{\rm{Forcing}}\,{\rm{comparison}}\,{\rm{parameter}}$$

$${\alpha }^{2}={\mu }_{c}/(\frac{m{U}_{HS}^{n-1}}{{H}^{n+1}}):\,{\rm{Dimensionless}}\,{\rm{Drag}}\,{\rm{parameter}}$$

Here, Γ is the forcing comparison parameter which compares the forcing due to pressure driven transport and due to electroosmotic transport. Thus, for Γ < 1, the electroosmotic transport becomes effective than the pressure driven transport, while for Γ > 1, the applied pressure gradient takes the dominating role on the underlying transport. For Γ = 1, both the forces become equally important on the transport process. The dimensionless drag parameter *α* signifies the variation in the Darcy drag in PEL. The change in the drag parameter *α* can be attributed to a change in the grafting density and size of the monomers. For higher values of *α*, the resistance to the flow increases due to dense polymeric distribution in the PEL. It should be noted that the PEL may act like a rigid boundary for higher values of *α*^13,48^.

#### Boundary conditions

To solve transport equations mentioned above [equations (12–15)], we use the following boundary conditions as discussed below^32^:

For potential distribution: In soft nanochannel, since the walls are uncharged, we consider the no flux condition or the Gauss boundary condition with zero surface charge density at the walls of the channel i.e. at $(\bar{y},\bar{z}=\pm \,1)$^49^. At the center $(\bar{y},\bar{z}=0)$ of the channel, the minima of the potential lead to the Neumann condition. At interface of the electrolyte and PEL, the potential (*ψ*) and the gradient of the potential (∇*ψ*) satisfies the continuity in potential distribution. Since the relative permittivity of electrolyte and PEL region does not change, we here consider the permittivity of both the PEL and electrolyte to be same^26^. Below we write the boundary conditions as discussed above.

16 $$\begin{array}{ll}{\frac{\partial {\bar{\psi }}_{p}}{\partial (\bar{y},\bar{z})}|}_{\bar{y},\bar{z}=\pm 1}=0 & :{\rm{Gauss}}\,{\rm{Boundary}}\,{\rm{condition}}\,{\rm{at}}\,{\rm{walls}}\\ {{\bar{\psi }}_{p}|}_{\bar{y},\bar{z}=(\pm 1)-\bar{d}}={{\bar{\psi }}_{e}|}_{\bar{y},\bar{z}=(\pm 1)-\bar{d}} & :{\rm{Potential}}\,{\rm{continuity}}\,{\rm{at}}\,{\rm{PEL}}/{\rm{EL}}\,{\rm{interface}}\\ {\frac{\partial {\bar{\psi }}_{p}}{\partial (\bar{y},\bar{z})}|}_{\bar{y},\bar{z}=(\pm 1)-\bar{d}}={\frac{\partial {\bar{\psi }}_{e}}{\partial (\bar{y},\bar{z})}|}_{\bar{y},\bar{z}=(\pm 1)-\bar{d}} & :{\rm{Induced}}\,{\rm{electric}}\,{\rm{field}}\,{\rm{continuity}}\,{\rm{at}}\,{\rm{PEL}}/{\rm{EL}}\,{\rm{interface}}\\ {\frac{\partial {\bar{\psi }}_{e}}{\partial (\bar{y},\bar{z})}|}_{\bar{y},\bar{z}=0}=0 & :{\rm{Symmetry}}\,{\rm{condition}}\,{\rm{at}}\,{\rm{the}}\,{\rm{center}}\,{\rm{of}}\,{\rm{the}}\,{\rm{channel}}\end{array}\}$$

For velocity distribution: For velocity distribution, we consider the no slip boundary condition at the walls of the channel, while at the center, due to symmetry, a Neumann condition is considered. At the PEL and electrolyte interface, we consider that the velocity and velocity gradient are equal in order to satisfy the continuity in the domain. It should be noted that the viscosity of the electrolytic solution is assumed to be the same in both the PEL and electrolyte regions^23^.

17 $$\begin{array}{cc}{{\bar{u}}_{p}|}_{\bar{y},\bar{z}=\pm 1}=0 & :{\rm{N}}{\rm{o}}\,{\rm{s}}{\rm{l}}{\rm{i}}{\rm{p}}\,{\rm{b}}{\rm{o}}{\rm{u}}{\rm{n}}{\rm{d}}{\rm{a}}{\rm{r}}{\rm{y}}\,{\rm{c}}{\rm{o}}{\rm{n}}{\rm{d}}{\rm{i}}{\rm{t}}{\rm{i}}{\rm{o}}{\rm{n}}\,{\rm{a}}{\rm{t}}\,{\rm{w}}{\rm{a}}{\rm{l}}{\rm{l}}{\rm{s}}\\ {{\bar{u}}_{p}|}_{\bar{y},\bar{z}=(\pm 1)-\bar{d}}={{\bar{u}}_{e}|}_{\bar{y},\bar{z}=(\pm 1)-\bar{d}} & :{\rm{V}}{\rm{e}}{\rm{l}}{\rm{o}}{\rm{c}}{\rm{i}}{\rm{t}}{\rm{y}}\,{\rm{c}}{\rm{o}}{\rm{n}}{\rm{t}}{\rm{i}}{\rm{n}}{\rm{u}}{\rm{i}}{\rm{t}}{\rm{y}}\,{\rm{a}}{\rm{t}}\,{\rm{P}}{\rm{E}}{\rm{L}}/{\rm{E}}{\rm{L}}\,{\rm{i}}{\rm{n}}{\rm{t}}{\rm{e}}{\rm{r}}{\rm{f}}{\rm{a}}{\rm{c}}{\rm{e}}\\ {{\bar{\tau }}_{p}|}_{\bar{y},\bar{z}=(\pm 1)-\bar{d}}={{\bar{\tau }}_{e}|}_{\bar{y},\bar{z}=(\pm 1)-\bar{d}} & :{\rm{S}}{\rm{h}}{\rm{e}}{\rm{a}}{\rm{r}}\,{\rm{s}}{\rm{t}}{\rm{r}}{\rm{e}}{\rm{s}}{\rm{s}}\,{\rm{c}}{\rm{o}}{\rm{n}}{\rm{t}}{\rm{i}}{\rm{n}}{\rm{u}}{\rm{i}}{\rm{t}}{\rm{y}}\,{\rm{a}}{\rm{t}}\,{\rm{P}}{\rm{E}}{\rm{L}}/{\rm{E}}{\rm{L}}\,{\rm{i}}{\rm{n}}{\rm{t}}{\rm{e}}{\rm{r}}{\rm{f}}{\rm{a}}{\rm{c}}{\rm{e}}\\ {\frac{{\rm{\partial }}{\bar{u}}_{e}}{{\rm{\partial }}(\bar{y},\bar{z})}|}_{\bar{y},\bar{z}=0}=0 & :{\rm{S}}{\rm{y}}{\rm{m}}{\rm{m}}{\rm{e}}{\rm{t}}{\rm{r}}{\rm{y}}\,{\rm{c}}{\rm{o}}{\rm{n}}{\rm{d}}{\rm{i}}{\rm{t}}{\rm{i}}{\rm{o}}{\rm{n}}\,{\rm{a}}{\rm{t}}\,{\rm{t}}{\rm{h}}{\rm{e}}\,{\rm{c}}{\rm{e}}{\rm{n}}{\rm{t}}{\rm{e}}{\rm{r}}\,{\rm{o}}{\rm{f}}\,{\rm{t}}{\rm{h}}{\rm{e}}\,{\rm{c}}{\rm{h}}{\rm{a}}{\rm{n}}{\rm{n}}{\rm{e}}{\rm{l}}\end{array}\}$$

The expression of shear stresses ${{\bar{\tau }}_{p}|}_{\bar{y},\bar{z}=(\pm 1)-\bar{d}}$ and ${{\bar{\tau }}_{e}|}_{\bar{y},\bar{z}=(\pm 1)-\bar{d}}$ in both the layers (PEL and electrolyte respectively) appearing in above equation takes the following form as written below:

For polyelectrolyte layer:

18 $${{\bar{\tau }}_{p}|}_{\bar{y},\bar{z}=(\pm 1)-\bar{d}}=[{({(\frac{\partial {\bar{u}}_{p}}{\partial \bar{y}})}^{2}+{(\frac{\partial {\bar{u}}_{p}}{\partial \bar{z}})}^{2})}^{\frac{n-1}{2}}]{\frac{\partial {\bar{u}}_{p}}{\partial (\bar{y},\bar{z})}|}_{\bar{y},\bar{z}=(\pm 1)-\bar{d}}$$

For electrolyte layer:

19 $${{\bar{\tau }}_{e}|}_{\bar{y},\bar{z}=(\pm 1)-\bar{d}}=[{({(\frac{\partial {\bar{u}}_{e}}{\partial \bar{y}})}^{2}+{(\frac{\partial {\bar{u}}_{e}}{\partial \bar{z}})}^{2})}^{\frac{n-1}{2}}]{\frac{\partial {\bar{u}}_{e}}{\partial (\bar{y},\bar{z})}|}_{\bar{y},\bar{z}=(\pm 1)-\bar{d}}$$

### Numerical Methods

In this analysis, we use our in-house developed finite difference code to solve the transport governing equations of the flow dynamics [equations (12–15)] for the present problem. Since we are focusing on the steady state, non-convective (low Reynolds number flow) flow of a Non-Newtonian fluid under the application of externally applied pressure gradient, we consider Gauss Seidel iterative method over the uniform grids of the computational domain to calculate the potential and velocity distributions. We discuss the details of this computational procedure as follows:

#### Computational domain

**Figure 2:**
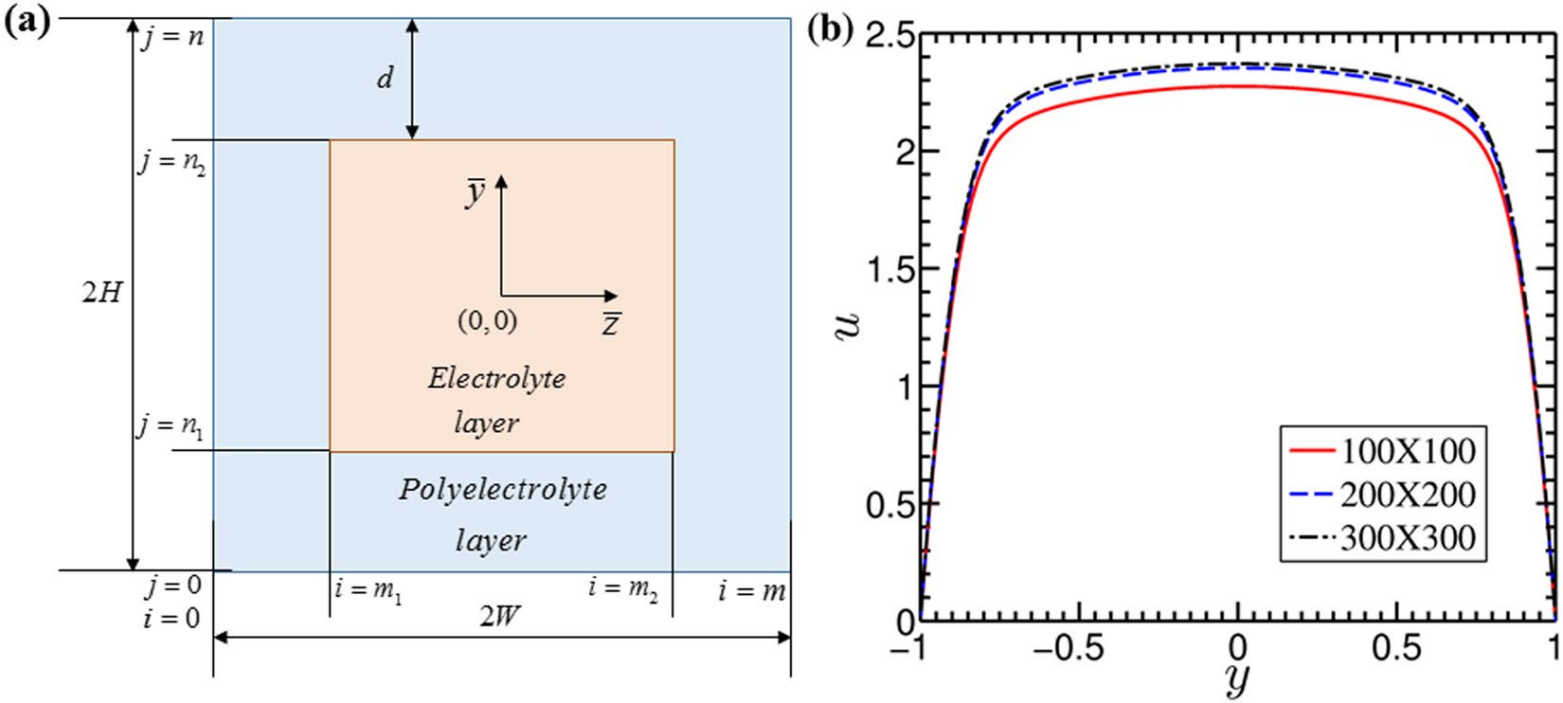
(**a**) *Computational domain*. Figure shows the schematic of the computational domain. The dimensions 2*W* and 2*H* are the width and height of the channel respectively. The center of the coordinate system is located at the center of the channel i.e., at $(\bar{z},\bar{y})=(0,0)$. The grid lines in *z* and *y* direction are denoted by *i*(0, *m*) and *j*(0, *n*) respectively. (**b**) *Grid dependence test*. Plot shows the variation in the velocity profiles with a change in the grid size. By observation, it can be concluded that the finer grid of the size 200 × 200 can be considered as the accurate one for the present numerical framework.

In Fig. 2(a), we show the computational domain of the present problem, while the coordinate system is attached at the center of the channel i.e., at $(\bar{z},\bar{y})=(0,0)$. We divide this domain in uniform grid of size *m*×*n*. The interface between the electrolyte layer and the PEL is depicted by the grid lines *i* = *m*_1_, *m*_2_ and *j* = *n*_1_, *n*_2_ in *z* and *y* directions respectively.

#### Solution procedure

We first calculate the induced potential *ψ* in both the electrolyte layer and polyelectrolyte layer using central difference approach. The discretized equations for the potential distribution [equations (12) and (13)] read as:

For electrolyte layer

20 $${\psi }_{i,j}^{t}=[(\frac{{\psi }_{i+1,j}^{t-1}+{\psi }_{i-1,j}^{t}}{{({\rm{\Delta }}z)}^{2}})+(\frac{{\psi }_{i,j+1}^{t-1}+{\psi }_{i,j-1}^{t}}{{({\rm{\Delta }}y)}^{2}})]/[{\kappa }^{2}+2(\frac{1}{{({\rm{\Delta }}z)}^{2}}+\frac{1}{{({\rm{\Delta }}y)}^{2}})]\,{\rm{where}}\,{\psi }_{i,j}\in {\psi }_{e}$$

For polyelectrolyte layer

21 $${\psi }_{i,j}^{t}=[{\kappa }_{p}^{2}+(\frac{{\psi }_{i+1,j}^{t-1}+{\psi }_{i-1,j}^{t}}{{({\rm{\Delta }}z)}^{2}})+(\frac{{\psi }_{i,j+1}^{t-1}+{\psi }_{i,j-1}^{t}}{{({\rm{\Delta }}y)}^{2}})]/[{\kappa }^{2}+2(\frac{1}{{({\rm{\Delta }}z)}^{2}}+\frac{1}{{({\rm{\Delta }}y)}^{2}})]\,{\rm{where}}\,{\psi }_{i,j}\in {\psi }_{p}$$

In above equations, Δ*y* and Δ*z* are the height and width of the each grid respectively. The superscript *t* of the *ψ*_*i*,*j*_ represents the present iteration level in the numerical calculation. Using these equations, we calculate the potential distribution across the channel cross section, which are then employed to calculate the velocity distribution of the fluid. As such, we use following set of discretized equations while performing the calculations mentioned above:For electrolyte layer

22 $${u}_{i,j}^{t}=[{F}_{{1}_{i,j}}^{t}+{F}_{{2}_{i,j}}^{t}-2{\rm{\Gamma }}+{g}_{i,j}^{t-1}{F}_{{3}_{i,j}}^{t}]/[2{g}_{i,j}^{t-1}{F}_{4}]\,\,{\rm{w}}{\rm{h}}{\rm{e}}{\rm{r}}{\rm{e}}\,{u}_{i,j}^{t}\in {u}_{e}$$

For polyelectrolyte layer

23 $${u}_{i,j}^{t}=[{F}_{{1}_{i,j}}^{t}+{F}_{{2}_{i,j}}^{t}-2{\rm{\Gamma }}+{g}_{i,j}^{t-1}{F}_{{3}_{i,j}}^{t}]/[2{g}_{i,j}^{t-1}{F}_{4}-{\alpha }^{2}]\,\,{\rm{where}}\,{u}_{i,j}^{t}\in {u}_{e}$$

In above equations, the functions ${F}_{{1}_{i,j}}^{t}\,{\rm{and}}\,{F}_{{2}_{i,j}}^{t}$ takes the following form as:

24 $${F}_{{1}_{i,j}}^{t}=[(\frac{{g}_{i+1,j}^{t-1}-{g}_{i-1,j}^{t-1}}{2{\rm{\Delta }}z})\cdot (\frac{{u}_{i+1,j}^{t-1}-{u}_{i-1,j}^{t}}{2{\rm{\Delta }}z})]+[(\frac{{g}_{i,j+1}^{t-1}-{g}_{i,j-1}^{t-1}}{2{\rm{\Delta }}y})\cdot (\frac{{u}_{i,j+1}^{t-1}-{u}_{i,j-1}^{t}}{2{\rm{\Delta }}y})]$$

25 $${F}_{{2}_{i,j}}^{t}=\frac{{\kappa }^{n+1}{\psi }_{i,j}^{t}}{{n}^{n}},\,{F}_{{3}_{i,j}}^{t}=(\frac{{u}_{i+1,j}^{t-1}+{u}_{i-1,j}^{t}}{{({\rm{\Delta }}z)}^{2}})+(\frac{{u}_{i,j+1}^{t-1}+{u}_{i,j-1}^{t}}{{({\rm{\Delta }}y)}^{2}}),\,{F}_{4}=\frac{1}{{({\rm{\Delta }}z)}^{2}}+\frac{1}{{({\rm{\Delta }}y)}^{2}}$$

Note that the function ${g}_{i,j}^{t-1}$ takes care of the non-linearity of the system, stemming from the rheology of the power-law model used in describing the constitutive behavior of the non-Newtonian fluid in this analysis. The function *g*(*z*, *y*) is defined as:

26 $$g(z,y)={[{(\frac{\partial u}{\partial z})}^{2}+{(\frac{\partial u}{\partial y})}^{2}]}^{\frac{n-1}{2}}$$

It is important to mention here that the term *g*_*i*,*j*_ in the above equations is calculated at previous iteration level to relieve the non-linearity of the system. However, for the conciseness in the presentation, we have not included the discretization of the function *g*_*i*,*j*_, since the calculation of *g*_*i*,*j*_ in the system changes with respect to boundary (forward difference or backward difference scheme) and the main domain (central difference scheme). It is important to mention here that, in this analysis, we have used Root Mean Square method to calculate the error in the calculation, while the least value of RMS error allowed in the calculation of potential and velocity distributions are 10^−8^ and 10^−12^ respectively.

#### Grid dependence test

**Figure 3:**
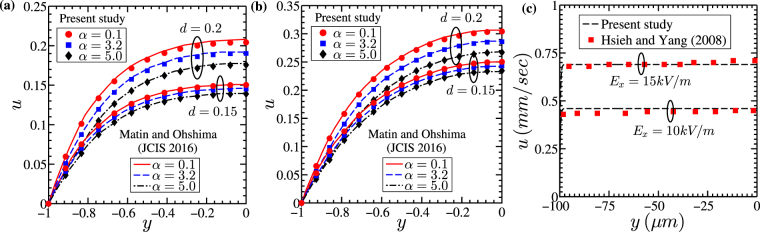
Plots showing the validation of present numerical calculation with the results reported by Matin and Ohshima^11^ and Hsieh and Yang^50^ respectively. *Theoretical validation*: A comparison of the velocity distribution across the channel cross section of the soft nanochannel obtained from present theoretical analysis vis-à-vis the reported results by the Matin and Ohshima^11^ is presented: for (**a**) Γ = 0 and (**b**) Γ = −0.1. *Experimental validation*: In Fig. (**c**), the velocity distributions obtained from the present numerical calculations are compared with the velocity profiles reported by Hsieh and Yang^50^ through experimental investigations. For validation results depicted in Fig. (c), we consider the value of PEL thickness *d* to be equal to 0.01 in order to mimic the flow physics of a rigid nanochannel, since Hsieh and Yang^50^ conducted experiments in a rigid channel. In both the cases, a fairly accurate match between the results of the present numerical modeling framework and the published results is witnessed.

In this study, we have considered finer grids to capture the variation in the electrostatics and the flow dynamics in the PEL region accurately. In Fig. 2(b), we show the effect of grid sizes on the results obtained from the present analysis. It is observed from Fig. 2(b) that, a change in fluid velocity becomes negligible as the grid size (*m* × *n*) change from 200 × 200 to 300 × 300. Accordingly, we have considered (*m* × *n*) = 200 × 200 for the present numerical calculations. Also, we would like to add here that the results obtained from the present numerical framework using (*m* × *n*) = 200 × 200 grid match well with the reported analytical as well as experimental results in the literature, depicted in Fig. 3(a–c). As such, these validation results further support that the results obtained from the present modeling framework are independent of grid sizes.

### Model Validation

We here consider dual benchmarking strategy. First, we validate the present numerical method with the results reported by Matin and Ohshima^11^. We show, in Fig. 3(a,b), the variation of flow velocity with dimensionless pressure gradient Γ = 0.0 and Γ = −0.1 respectively, obtained for different values of *α* and *d* as mentioned in the figures. While validating our model with the results of Matin and Ohshima^11^, the width (*W*) of the channel is considered to be much higher than its height (*H*). Note that the values of *α* and *d* considered in this study are in compliance with those as chosen by Matin and Ohshima^11^.

The dimensionless parameters taken for this validation are: *n* = 1, *κ*_*p*_ = 2.0, *κ* = 4.0. The results obtained from the present theoretical analysis show a good match with the reported results of Matin and Ohshima^11^. Second, we also take an effort, in Fig. 3(c), to validate the present theoretical analysis with the experimental results reported by Hsieh and Yang^50^ for two different values of axial electric field *E*_*x*_ = 15 *kV*/*m* and *E*_*x*_ = 10 *kV*/*m*. While validating our analysis with experimental results reported by Hsieh and Yang^50^, we shifted the PEL-electrolyte interface to the walls of the channel by taking PEL thickness *d* = 0.01 (equivalent to 1 nm), keeping other parameters same as that of considered by Hsieh and Yang^50^ and given as: $\kappa =1.06\,{{\rm{nm}}}^{-1}$, ${\kappa }_{p}=0.883\,{{\rm{nm}}}^{-1}$, $\varepsilon =644.47\times {10}^{-2}\,{{\rm{Fm}}}^{-1}$, $\eta =803.4\times {10}^{-6}\,{\rm{Pa}}.\,\sec$, *h* = 100 *μm*, *n* = 1, *k* = 2 × 10^−9^ *m*^2^ (permeability of the PEL). The plots are drawn at *z* = 0. We find from Fig. 3(c) a fairly accurate match between the present theoretical and experimental results.

## Results and Discussions

Imposing the boundary conditions mentioned in equations (16) and (17), we solve the governing transport equations [equation (12–15)] for the potential and velocity distribution using our in-house finite difference code. We discuss several results obtained from this analysis in the forthcoming sections systematically. Unless specified otherwise, referring to the typical values as report in the literature^11^, we consider the following set of parameters as: *κ* = 20, *κ*_*p*_ = 10, *α* = 2.5, Γ = −0.5, *d* = 0.2 for this analysis.

### Potential and velocity distribution

**Figure 4:**
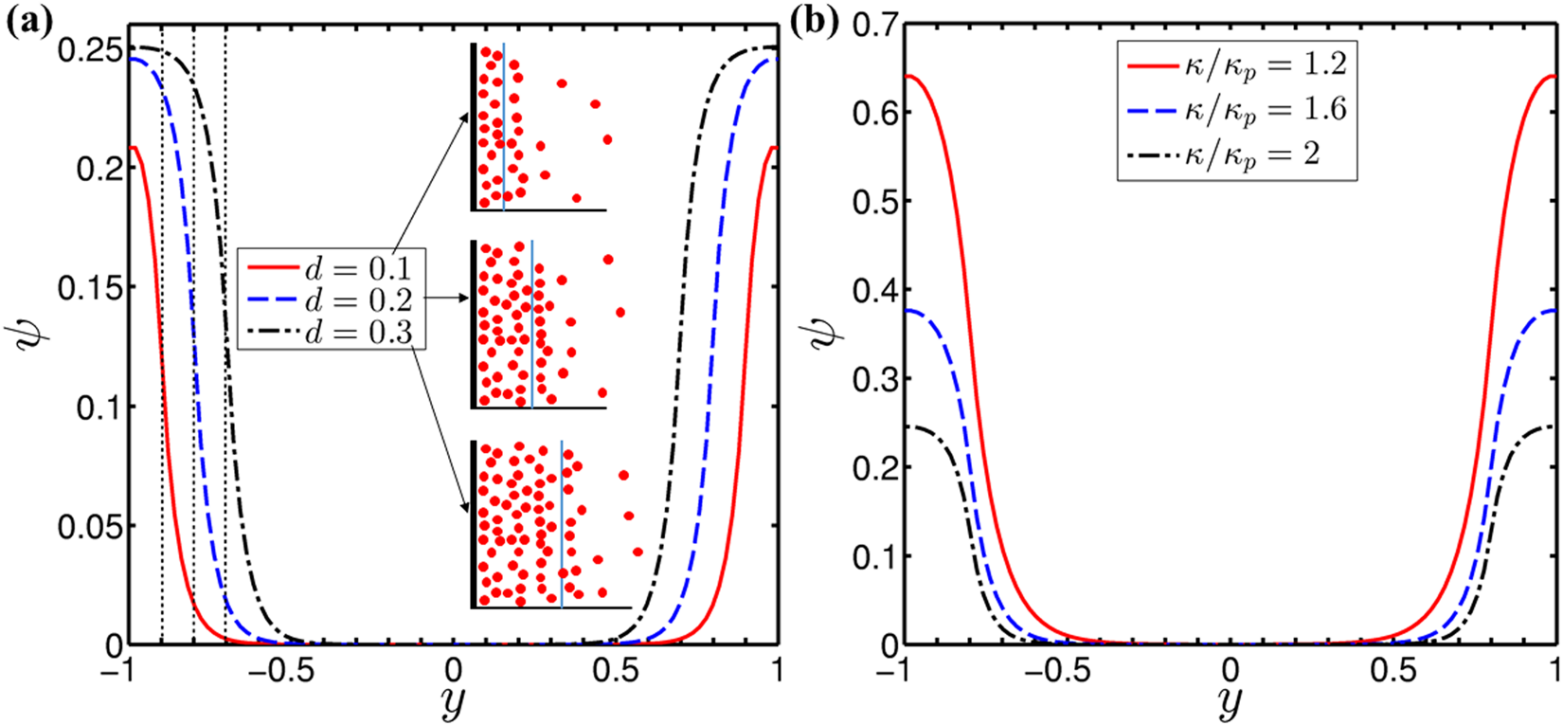
Plots depict the potential distribution in channel at *z* = 0 for (**a**) three different values of dimensionless PEL thickness as: *d* = 0.1, 0.2 and 0.3 and (**b**) for three different values of Debye-Huckel ratio *κ*/*κ*_*p*_  =  1.2, 1.6 and 2. We also show a charge distribution for variation of PEL thickness in the inset of (**a**). For (a), we consider other parameters as: *κ* = 20, *κ*_*p*_ = 10, while for (**b**) those are: *κ*_*p*_ = 10, *d* = 0.2.

To understand the variant behavior of the flow dynamics inside the soft narrow fluidic channel, the relative strength of the volumetric body forces is one of the important point to be looked at critically. In soft narrow fluidic channel, an inclusion of PEL layer on the walls of the channel and different volumetric body forces fetch a fascinating flow physics. In order to comprehend the variance in the electroosmotic body force in the soft narrow fluidic channels for different PEL parameters *viz*., induced drag parameter (*α*), PEL thickness (*d*) and the ratio of Debye-Huckel parameter (*κ*/*κ*_*p*_), a variation in the potential distribution across the channel cross section needs to be focused at once, albeit it was reported by many researchers earlier^23,26^. Taking this aspect into consideration, first we report, in Fig. 4, the variation in the potential distribution in the channel. As such, the variation of potential distribution delineated in Fig. 4 will be required to support the non-intuitive results described in the subsequent sections.

**Figure 5:**
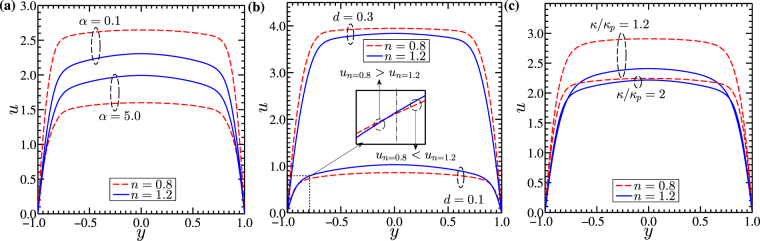
Above figure depicts the variation in the velocity distribution at z  =  0 for shear-thinning (*n* = 0.8) and shear-thickening fluid (*n* = 1.2). We analyze the effect of (**a**) induced drag parameter (*α*), (**b**) non-dimensional PEL thickness (*d*) and (**c**) Debye-Huckel parameter ratio (*κ*/*κ*_*p*_) respectively on the velocity distribution with all other parameters considered to be same. The inset of (b) shows that near the walls of the channel: ${u}_{n=0.8} > {u}_{n=1.2}$ while near the center of channel it shows ${u}_{n=0.8} < {u}_{n=1.2}$ for a case of *d* = 0.1. We consider the other parameters as: for (**a**) *κ* = 20, *κ*_*p*_ = 10, Γ = −0.5, *d* = 0.2; for (**b**) *κ* = 20, *κ*_*p*_ = 10, *α* = 2.5, Γ = −0.5; and for (**c**) *κ*_*p*_ = 10, *α* = 2.5, Γ = −0.5, *d* = 0.2.

In Fig. 4(a,b), we show the effect of PEL thickness (*d*) and Debye-Huckel ratio (*κ*/*κ*_*p*_) respectively on the potential distribution. We consider different values of PEL thickness (*d*) as 0.1, 0.2 and 0.3 in Fig. 4(a), while in Fig. 4(b), we take three different values of ratio of Debye-Huckel parameter (*κ*/*κ*_*p*_) as 1.2, 1.6 and 2. Having a closer look at Fig. 4, we observe that the potential in the channel gets enhanced with the increasing value of *d* from 0.1 to 0.3 as well as with the decreasing magnitude of *κ*/*κ*_*p*_ from 2 to 1.2. In an effort to figure out the physical explanation behind this variation, we invoke to the variation of the charge distribution in the PEL. A variation in the softness of the narrow fluidic channel is mainly attributed to the charge distribution in the PEL. With an increment in the PEL thickness (or equivalently with a reduction in the ratio of Debye-Huckel parameter), the charge density in the PEL increases due to a relatively stronger interaction of the PEL ions with the ions of the electrolyte layer. Such increment in the charge concentration in the PEL leads to an enhancement in the induced potential in the PEL and that too in the electrolyte layer according to Poisson-Boltzmann potential distribution^2,32^. This phenomenon eventually gives rise to an enrichment in the potential as observed in Fig. 4(a,b) with a variation in the PEL thickness (*d*) from 0.1 to 0.3 and ratio of Debye-Huckel parameter (*κ*/*κ*_*p*_) from 2.0 to 1.2. Taking this variation of the potential distribution (delineated in Fig. 4) into account, we next move to discuss the variation of velocity distribution in the channel as depicted in Fig. 5(a–c).

**Figure 6:**
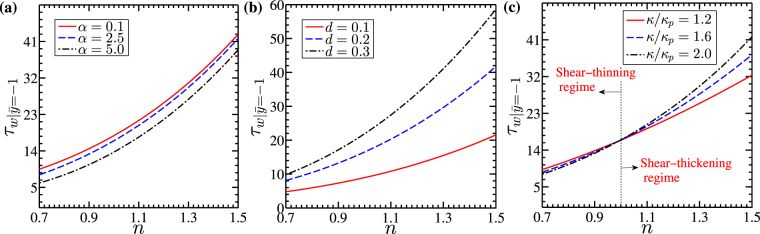
Figure shows the variation in the wall shear stress at location *y* = −1,*z* = 0 with power law behavior for three different cases of (**a**) induced drag parameter *α* = 0.1, 2.5 and 5, (**b**) non-dimensional PEL thickness *d* = 0.1, 0.2 and 0.3 and (**c**) Debye Huckel parameter ratio *κ*/*κ*_*p*_  =  1.2, 1.6 and 2 respectively. The other parameters considered are: for Fig. 6(a) *κ* = 20, *κ*_*p*_ = 10, Γ = −0.5, *d* = 0.2 for Fig. 6(b) *κ* = 20, *κ*_*p*_ = 10, *α* = 2.5, Γ = −0.5 for Fig. 6(c) *κ*_*p*_ = 10, *α* = 2.5, Γ = −0.5, *d* = 0.2.

In Fig. 5(a–c), we show the variation in velocity distribution in the channel for three different parameters *viz*., induced drag parameter (*α*), dimensionless PEL thickness (*d*) and the ratio of Debye-Huckel parameter (*κ*/*κ*_*p*_). For all the variations depicted in Fig. 5(a–c), we consider two different power-law indices *n* = 0.8 and *n* = 1.2^40^. In Fig. 5(a–c), we observe a plug like profile of a flow velocity in the channel for all values of *α*, *d* and *κ*/*κ*_*p*_ considered. In fact, this observation holds true for both the shear-thinning (*n* = 0.8) and shear-thickening (*n* = 1.2) fluids. The parameters governing the characteristics of PEL affect the velocity distribution and its magnitude by different aspects as clearly seen in Fig. 5(a–c). The frictional drag in the PEL impacts the underlying transport following the same nature as the Darcy friction drag does on the underlying transport through a porous media. An increment in the polymer grafting density resulting from an increasing value of drag parameter (*α*) enhances the Darcy drag, which in effect reduces the velocities in the channel for both values of power-law index considered in Fig. 5(a). The associated wall shear stress for a relatively higher value of drag parameter *α*(=5) is also less for all the values of power law index considered (n = 0.7 to 1.5) as can be seen in Fig. 6(a). As such, the lesser magnitude of wall shear stress for *α* = 5 as seen in Fig. 6(a) conforms to the corresponding velocity magnitude in the channel in Fig. 5(a). Also, we observe from Fig. 5(a) that the velocity for the shear-thinning fluid (*n* = 0.8) is higher than that of the shear-thickening fluid (*n* = 1.2) for *α* = 0.1, while for *α* = 5, a reverse scenario of higher velocity attained by the shear-thickening fluid (*n* = 1.2) is observed. We attribute this observation to the effect of viscous drag on the underlying flow dynamics. Since the apparent viscosity of class of shear-thinning fluids (*n* < 1) is less, the viscous resistance offered to the flow field of a shear-thinning fluid (*n* < 1) becomes lesser as compared to that of the shear-thickening fluid (*n* > 1) for a given strength of driving force being applied to make the flow occur. Consequently, a relatively lesser viscous resistance makes the flow velocity of shear-thinning fluid (*n* = 0.8) to be higher as reflected in Fig. 5(a) for *α* = 0.1. However, a closer look at Fig. 5(a) further reveals that, the reduction in the flow velocity for a higher value of *α*(=5) becomes relatively higher for the flow of a shear-thinning fluid (*n* = 0.8). We attribute this observation to the effect of viscous resistance, since viscous resistance experienced by the flow of a shear-thinning fluid (*n* = 0.8) is lesser than that of the shear-thickening fluid (*n* = 1.2).

The effect of PEL on the underlying transport can be better understood by varying the PEL thickness as delineated in Fig. 5(b). As such, a change in the thickness of PEL (thickness of the soft layer) in the channel brings in a noticeable change on the underlying electrohydrodynamics as seen in Fig. 5(b). An increment in the PEL thickness augments the electrical forcing in fluid domain on account of an enhanced ionic interaction between PEL and electrolyte layer^32^. Therefore, the higher PEL thickness as realized by a higher value of *d*(=0.3) leads to a high velocity in the channel as depicted in Fig. 5(b), attributed primarily to the combined influences of increased potential (see Fig. 3a) and higher electrical body force rendered by a relatively higher value of the PEL thickness. The higher velocity in the channel leads to an increment in the wall shear stress for higher PEL thickness as confirmed in Fig. 6(b).

Next, we describe, in Fig. 5(c), the variation of flow velocity influenced by the ratio of Debye-Huckel parameter. It should be mentioned in this context here that we keep, in this analysis, the value of *κ*_*p*_ fixed at 10, while the magnitude of *κ* is varied from 12 to 20 essentially to obtain the variation in the Debye-Huckel parameter ratio in the range of 1.2 to 2.0^32^. With an increment in *κ*/*κ*_*p*_ from 1.2 to 2.0, the magnitude of potential distribution in the channel decreases as depicted in the Fig. 4(b). For a fixed value of *d* = 0.2, the potential in the channel is seen to be higher for *κ*/*κ*_*p*_ = 1.2, largely attributed to a comparatively higher concentration of PEL ions, leading to a rise in the softness offered by the PEL to electrolyte^32^. Therefore, the velocity magnitude for *κ*/*κ*_*p*_ = 1.2 becomes higher than that of *κ*/*κ*_*p*_ = 2.0 as witnessed in Fig. 5(c).

We would like to discuss other important points in the context of Fig. 5(b) as follows: One may find from Fig. 5(b) that the fluid velocity for shear-thinning fluid is higher for particular case of *d* = 0.3, whereas for the remaining case of *d* = 0.1, the velocity for shear-thickening fluid (*n* = 1.2) is higher than that of the shear-thinning fluid (*n* = 0.8). It may be mentioned here that the case of *d* = 0.1 nearly mimics a rigid narrow fluidic channel due to very small thickness of the PEL and hence, the electrical forcing is remaining effective only near the walls of channel. Due to this, the electrical body force experienced by the shear-thinning fluid (*n* = 0.8) is higher than the shear-thickening fluid (*n* = 1.2) in the near wall region, since the apparent viscosity and so is the viscous resistance to the flow field becomes lesser for the flow of a shear-thinning fluid. As such, upon experiencing a relatively higher magnitude of electrical body force, the velocity of the shear-thinning fluid becomes higher than that of shear-thickening fluid near the walls as shown in the inset of Fig. 5(b). On the other hand, because of the higher viscosity of the shear-thickening fluid (*n* = 1.2), the net momentum due to electrical forcing gets transported to a relatively larger lateral extent of the channel, which in turn leads to a higher velocity of the shear-thickening fluid (*n* = 1.2) in the center of the channel (for *d* = 0.1) as witnessed in Fig. 5(b). The higher momentum due to electrical forcing stemming from the EDL being formed upon ionic interactions between PEL and electrolyte layer give rise to a relatively higher velocity for the shear-thinning fluid (*n* = 0.8) in the soft narrow fluidic channel as seen in Fig. 5(b). We would like to discuss another important point in the context of Fig. 5(b) as follows: although the fluid velocity in the region closer to the walls of the channel is higher for the shear-thinning fluid (*n* = 0.8) for both the values of *d* considered (see inset of Fig. 5(b)), the magnitude of velocity in the bulk region is seen to be higher for shear-thickening (*n* = 1.2) fluid (almost comparable for *d* = 0.3) attributed largely to the rheology driven modifications in the viscous resistance as modulated by the electrical forcing. For the case of *d* = 0.3, since the electrical forcing acting on the fluid mass originating from the EDL being formed upon interaction of PEL and electrolyte layer becomes relatively stronger, the higher rate of momentum transport even by the shear-thinning fluid (although viscosity is less) makes the fluid velocity to be almost equal with the velocity attained by the shear-thickening (*n* = 1.2) fluid in the bulk region. On the other hand, for *d* = 0.1, the net electrical forcing applied on the fluid mass becomes relatively lesser (the ionic concentration between the PEL and electrolyte layer is less for *d* = 0.1) and hence, the rate of momentum transport by the shear-thinning fluid is not comparable with that of the shear-thickening fluid (momentum transport by the shear-thickening fluid is always higher on account of a higher viscosity of this class of fluid), leading to a lesser velocity of the shear-thinning fluid in the bulk region as seen in Fig. 5(b).

### Effect of soft layer on wall shear stress

The grafting of PEL in the narrow fluidic channel alters the underlying flow dynamics and the rate of volumetric transport through the channel following an alteration in shear stress. We take an effort, in Fig. 6(a–c), to see the variation of wall shear stress versus power-law indices obtained for different values of drag parameter *α*(=0.1, 2.5 and 5), PEL thickness *d*(=0.1, 0.2 and 0.3) and Debye-Huckel parameter ratio *κ*/*κ*_*p*_(=1.2, 1.6 and 2) respectively.

We observe that the wall shear stress in the soft narrow fluidic channel for all the parametric variations (*viz*. $\alpha ,d\,{\rm{and}}\,\kappa /{\kappa }_{p}$) considered in Fig. 6(a–c) gets enhanced with an increment in the power law index i.e. with a change in fluid rheology from shear-thinning (*n* < 1) to the shear-thickening (*n* > 1) behavior. We attribute this variation to an increment in the apparent viscosity of the fluid, which increases with an increment in the non-Newtonian behavior of the fluid. As the power-law index varies from 0.7 to 1.5, the apparent viscosity of the fluid increases, leading to an increment in the shear stress at the wall as depicted in Fig. 6. The plots depicted in Fig. 6(a) reflect the effect of change in the PEL drag parameter on the variation of wall shear stress. With an increment in the drag parameter *α* from 0.1 to 5, the wall shear stress reduces due to the reduction in fluid velocity near the walls of the channel. Note that a reduction in velocity with increasing *α* is confirmed in Fig. 5(a). However, due to increasing viscosity of the non-Newtonian fluid with increasing value of power-law index (*n* = 0.7 to 1.5), the wall shear stress in the channel enhances more noticeably. Next we look at Fig. 6(b), which shows the variation of wall shear stress obtained for different values of $d(=0.1,0.2\,{\rm{and}}\,0.3)$. It may be mentioned here that with the increasing value of PEL thickness (*d*) from 0.1 to 0.3, the softness of the channel increases, which in effect leads to a rise in the fluid velocity as confirmed in Fig. 5(b). Such enhancement in the fluid velocity with a higher value of *d* as witnessed in Fig. 5(b) together with the stronger EDL effect leads to a higher velocity gradient in the channel and resulting in a higher wall shear stress as confirmed in Fig. 6(b). It is worth mentioning here that the wall shear stress for higher PEL thickness (*d* = 0.3) grows exponentially with power-law index as clearly seen in Fig. 6(b). We further attribute this observation to a relatively higher apparent viscosity of the class of shear-thickening fluids (*n* > 1) than the shear-thinning fluids (*n* < 1).

Further, an inversion in the shear stress profiles is seen in the variation obtained for three different values of *κ*/*κ*_*p*_(=1.2, 1.6 and 2.0), while varying the power law index from 0.7 to 1.5 as delineated in Fig. 6(c). A closer look at Fig. 6(c) discloses that the inversion occurs at *n* = 1(Newtonian fluid). As mentioned before, for *κ*/*κ*_*p*_ = 1.2, due to a comparatively higher surface potential (see Fig. 4(b)) the velocity of shear-thinning fluid (*n* < 1) becomes higher than the shear-thickening fluid (*n* > 1), which results in a higher velocity gradient near the walls for the underlying transport of shear-thinning fluid. This higher velocity gradient gives rise to a higher wall shear stress for the flow of shear-thinning fluid for *κ*/*κ*_*p*_ = 1.2 as one may verify from Fig. 6(c). On the contrary, for the case of *κ*/*κ*_*p*_ = 2, a reverse scenario of increasing wall stress for shear-thickening fluid (*n* > 1) is witnessed in Fig. 6(c) as well. In the region of *n* > 1, due to comparatively low ionic concentration in PEL and low surface potential, the velocity of the shear-thickening fluid is higher than the shear-thinning fluid for the case of *κ*/*κ*_*p*_ = 2, thus results in a higher wall shear stress for the shear-thinning fluid as confirmed in Fig. 6(c).

### Volumetric flow rate

An intend of different types of annexations in the nanofluidic devices/systems ultimately targets to achieve an enhancement in the flow rate as well as its control through these systems/devices, essentially to meet the demand of the micro-total-analysis-systems (*μ*TAS) or micro-electro-mechanical-systems (MEMS). Accordingly, we here present the variation of flow rate in the soft narrow fluidic channel for different values of parameters, which govern the hydrodynamics through PEL such as dimensionless PEL thickness (*d*) and drag parameter (*α*). Below, we write the explicit expression of the dimensionless volumetric flow rate as:

27 $$Q={\int }_{-1}^{1}{\int }_{-1}^{1}\bar{u}(\bar{z},\bar{y})d\bar{z}d\bar{y}$$

Here, we obtain the variations in the volumetric flow rate in the channel by varying the Debye-Huckel parameter of electrolyte layer (*κ*) from 12 to 20 and power-law index (*n*) from 0.7 to 1.5 (shear-thinning to shear-thickening nature). As mentioned before, a fixed value of Debye-Huckel parameter for PEL (*κ*_*p*_ = 10) is considered to obtain the desired variations, which in essence makes Debye-Huckel parameter ratio (*κ*/*κ*_*p*_) to vary from 1.2 to 2 in this analysis. Below we discuss the variations of flow rate with PEL thickness *d* and drag parameter *α* systematically.

#### Effect of PEL thickness

**Figure 7:**
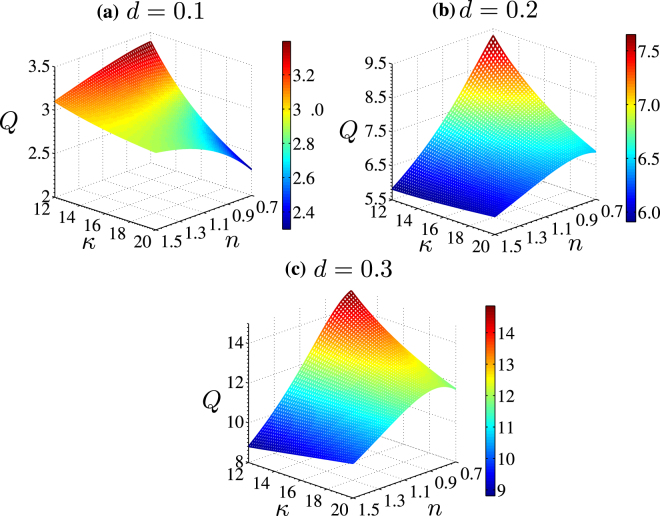
Plots depict the effect of a change in the PEL thickness on the volumetric flow rate in the soft narrow fluidic channel. We vary dimensionless PEL thickness (*d*) from 0.1, 0.2 and 0.3 while the variation in the Debye-Huckel parameter and power law index remains the same as mentioned above. The other parameters considered are: *κ*_*p*_ = 10, *α* = 2.5, Γ = −0.5.

Figure 7(a–c) represent the surface plot (2*D*) of the variation of volumetric flow rate versus the Debye-Huckel parameter of electrolyte layer (*κ*) and power law index (*n*), obtained for three dimensionless values of PEL thickness $d=0.1,0.2\,{\rm{and}}\,0.3$ respectively. It is worth mentioning here that an increment in the PEL thickness enhances the charge concentration and so as the potential (see Fig. 4(a)) in both the PEL as well as electrolyte layer. Since an increment in the charge density leads to an augmentation in the electroosmotic body force in both PEL and electrolyte layer, the higher flow velocity in the soft narrow fluidic channels is observed for higher PEL thickness as confirmed in Fig. 5(b). Accordingly, an enhancement in the flow rate through the channel with an increment in PEL thickness from *d* = 0.1 to 0.3 as observed in Fig. 7(a–c), signifies the effect of the magnitude of electrical forcing, which increases with an increment in the PEL thickness, on the underlying transport while all other parameters remain unaltered. In fact, with an increment in *d*, the cumulative effect of the electrical body force originating from the EDL being formed upon ionic interaction between PEL and electrolyte layer, enhances the flow velocity for both the shear-thinning and shear-thickening fluids, leading to an enhancement in flow rate through the channel as apparent from Fig. 7(a–c). In Fig. 7(a), it is observed that, for lower values of *κ*(=12), the flow rate in the channel is higher for shear-thinning fluid (*n* < 1) than that of shear-thickening fluid (*n* > 1), whereas for higher values of *κ*(=20), the opposite scenario is observed. This observation can be attributed to the conglomeration of the two phenomena, one is variation of the electrical forcing with *κ* and second one is the softness induced modification of the rheology of the non-Newtonian fluid. Since the viscous resistance applied to shear-thinning fluid (*n* < 1) is less as compared to the shear-thickening fluid (*n* > 1), the higher magnitude of electrical body force for lower value of *κ*(=12) enhances the transport of shear-thinning fluid (*n* < 1) in the channel (as compared to the shear-thickening (*n* > 1) fluid), leading to higher flow rate of shear-thinning fluid (*n* < 1) as observed in Fig. 7(a). However, for higher values of *κ*(=20) in case of *d* = 0.1, the smaller magnitude of electrical body force implies a case of rigid nanochannel as elaborated before in the context of Fig. 5(b). For thinner PEL (*d* = 0.1) and for higher values of *κ*(=20), the shear-thickening fluid (*n* > 1) shows higher velocity than that the shear-thinning fluid (*n* < 1) due to higher transverse momentum exchange phenomena, approximately mimicking a rigid nanochannel case for *d* = 0.1 (see Fig. 5(b)). However, in Fig. 7(b,c), we do not observe such phenomena due to higher effect of the softness, induced by the increasing values of *d* = 0.1−0.3.

Having a closer look at Fig. 7(b,c), which depict the flow rate variation for *d* = 0.2 and 0.3 respectively, one may find that the flow rate for the shear-thinning fluid becomes higher in comparison to the shear-thickening fluid. To make a comment on this observation, it may be mentioned here that, with an increment in the PEL thickness (*d* = 0.2,0.3), the electroosmotic body force increases due to a relatively stronger ionic interaction of PEL and electrolyte layer. To be precise, because of the higher electrical body force, the rate of momentum transport by the shear-thinning fluid makes the flow velocity of these kinds of fluids to be either comparable $({\rm{for}}\,d=0.2)$ or even higher $({\rm{for}}\,d=0.3)$ than that of the shear-thickening fluid. Thus, the higher flow velocity gained by the shear-thinning fluid leads to a higher flow rate in the channel for $d=0.2\,{\rm{and}}\,0.3$ as evident in Fig. 7(b,c).

#### Variation in drag parameter

**Figure 8:**
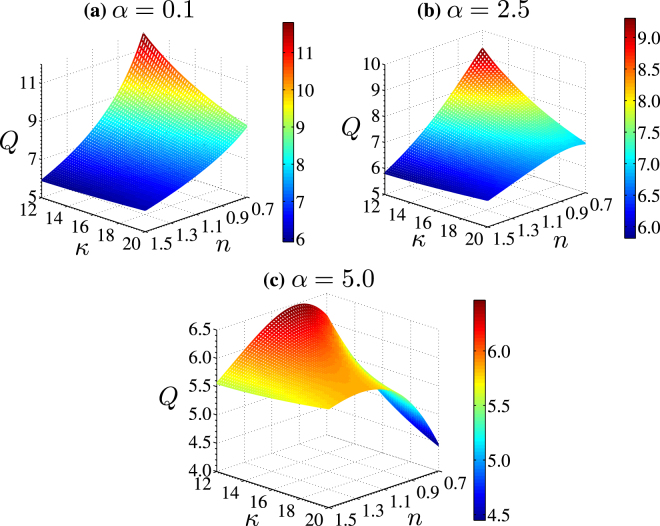
Plots depict the variation in the volumetric flow rate vs the power-law index (*n*) from 0.7 to 1.5 and electrolyte Debye-Huckel parameter (*κ*) from 12 to 20. The variation is carried out for three different cases of induced drag parameter (*α*) ranging from 0.1, 2.5 and 5. The other parameters considered are: *κ*_*p*_ = 10, Γ = −0.5, *d* = 0.2.

In Fig. 8(a–c), we delineate the effect of drag parameter (*α*) on the variation of volumetric flow rate in the channel. Note that we have considered three different values of $\alpha (=0.1,2.5\,{\rm{and}}\,5)$ for Fig. 8(a–c) respectively, while other parameters considered for plotting the figures are: *κ*_*p*_ = 10, Γ = −0.5, *d* = 0.2. The chosen values of $\alpha (=0.1,2.5\,{\rm{and}}\,5)$ considered in obtaining the present figures mimic the situation of an increment in the induced drag due to increased polymer density or grafting density of PEL^32^.

The variation of the drag in the PEL is essentially a manifestation of either the change in the porosity or the permeability of the porous structure inside the PEL matrix for which the dimensional drag parameter *μ*_*c*_ is written as *η*/*K*^51^, where *η* is viscosity of fluid and *K* is the permeability of the porous structure due to polymer density in the PEL. So with an increment in polymer or grafting density, the permeability of PEL decreases, which leads to a reduction in the fluid velocity in the PEL. In fact, a reduction in the flow velocity in PEL, accounting the exchange of transverse momentum phenomenon, further leads to a reduction of velocity in the electrolyte domain as well. As a consequence of this reduction in flow velocity in both the layers, we observe a reduction in the flow rate with an increment in the drag parameter from *α* = 0.1 to 5 as delineated in the Fig. 8(a–c). It is observed from Fig. 8(a) that the flow rate for small value of drag parameter *α* = 0.1 increases with lowering the Debye-Huckel parameter of electrolyte layer *κ* from 20 to 12 (or equivalently with a reduction in *κ*/*κ*_*p*_ from 2 to 1.2), while a change in power-law index from 1.5 to 0.7 leads to an increment in flow rate as well. However, a closer look at Fig. 8(a–c) reveals that, an increment in the drag parameter from *α* = 0.1 to 5 reduces the flow rate in the channel for shear-thinning fluid (*n* < 1) appreciably, whereas the variation of flow rate of the shear-thickening fluid (*n* > 1) with a change in *α* in the range considered for plotting the present figures is not significant. For the flow through a porous channel, the shear-thinning fluid is more susceptible to the change in the drag parameter (either increase or decrease) than the shear-thickening fluid, attributed primarily to the relatively lesser apparent viscosity inherent with the class of shear-thinning fluid (*n* < 1).

### Softness induced enhancement in net throughput

**Figure 9:**
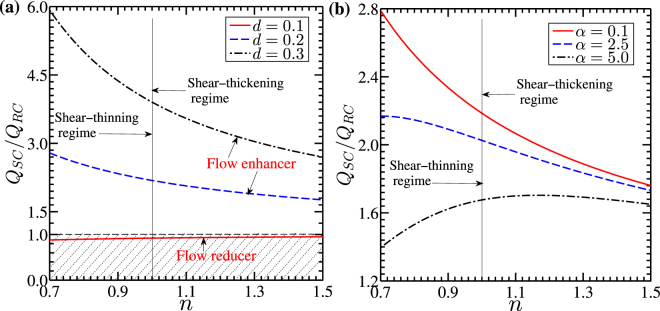
Plots show the variation of the flow rate ratio *Q*_*SC*_/*Q*_*RC*_ versus power-law index *n* (varies from 0.7 to 1.5). *Q*_*SC*_ and *Q*_*RC*_ are the volumetric flow rates in the soft and rigid nanochannel respectively. (**a**) Plots depict the variations of *Q*_*SC*_/*Q*_*RC*_ for different values of PEL thickness *d* = 0.1, 0.2 and 0.3 for *α* = 0.1 and (**b**) the variations in *Q*_*SC*_/*Q*_*RC*_ for different values of drag parameter *α* = 0.1, 2.5 and 5, obtained for *d* = 0.2 are depicted. The other parameters chosen for plotting above figures are: *κ* = 12, *κ*_*p*_ = 10 and Γ = −0.5. A continual decreasing trend of *Q*_*SC*_/*Q*_*RC*_ vs. *n* is seen for all the values of PEL thickness (*d*) considered. For a relatively higher value of *α*(=5), the flow rate ratio (*Q*_*SC*_/*Q*_*RC*_) shows an increasing trend in the shear-thinning regime, while a decreasing trend is witnessed in the shear-thickening regime.

We here discuss about the flow rate enhancement in the soft nanochannel compared to the rigid nanochannel. In doing so, we show in Fig. 9(a,b), the variation of the flow rate ratio (*Q*_*SC*_/*Q*_*RC*_), defined as the ratio of flow rate in the soft nanochannel (*Q*_*SC*_) to the flow rate in rigid nanochannel (*Q*_*RC*_), with a change in power-law index from 0.7 to 1.5, obtained for different values of *d* and *α* respectively. It may be mentioned in this context here that for plotting the variation of flow rate in rigid nanochannel (*Q*_*RC*_) in Fig. 9(a,b), we have considered the thickness of the PEL (*d*) to be 0.01, essentially to mimic the underlying transport through a rigid nanochannel.

It is observed from Fig. 9(a,b) that the ratio *Q*_*SC*_/*Q*_*RC*_ is always higher than unity for all the values of *d* and *α* considered, except for the case of *d* = 0.1 in Fig. 9(a). As such, this observation holds true for the range of *n* undertaken in the plotting the present figures. Grafting of polyelectrolyte layer at the walls of soft nanochannel makes the induced potential (*ψ*) to be higher than that of the rigid nanochannel for all values of power-law index, leading to a comparatively stronger electroosmotic body force being applied on the fluid mass in the EDL. In such cases, the soft nanochannel can be called as the flow enhancer, except for the case of *d* = 0.1. It should be noted here that the magnitude of electrical forcing in such cases (*d* ≤ 0.1) is very less as the PEL thickness is very small as compared to the other cases shown in Fig. 9(a,b). Furthermore, for *d* ≤ 0.1, the friction drag provided by the PEL to the fluid leads to a reduction in flow velocity for all classes of fluids (albeit reduction is higher for shear-thinning fluid) in Fig. 7. Thus, lesser electroosmotic force together with the frictional drag reduces the flow velocity, leading to a reduction in net throughput as compared to the rigid channel as seen in Fig. 9(a). Hence, except *d* = 0.1, we observe an enhancement in net throughput for all the values of *d* and *α* considered in this analysis, calling itself a flow enhancer region.

In Fig. 9(a), we observe a decreasing trend of *Q*_*SC*_/*Q*_*RC*_ with a change in the power-law index from 0.7 to 1.5, for *d* = 0.2 and *d* = 0.3, attributed primarily to the effect of the change in the fluid rheology from shear-thinning (*n* < 1) to the shear-thickening behavior of the fluid (*n* > 1). Since, the viscous resistance for the shear-thinning fluid is lesser than that of shear-thickening fluid, the flow rate obtained for shear-thinning fluid is higher than that of shear-thickening fluid, as witnessed in Fig. 7(a–c). In fact, the observations reflected in Fig. 7(a–c) get further verified in Fig. 9(a), where we find a relatively higher flow rate enhancement of shear-thinning fluid than that of the shear-thickening fluid. Such region of the enhancement in the flow rate for *d* ≥ 0.1 can be characterized as the flow enhancer. However, the region of *d* ≤ 0.1 can be called as the flow reducer since the flow of the non-Newtonian fluid (for all values of *n* = 0.7–1.5) is less than that of rigid nanochannel case (*Q*_*SC*_ < *Q*_*RC*_). Apart from this, in Fig. 9(a), we observe that *Q*_*SC*_/*Q*_*RC*_ increases with an increment in the PEL thickness from 0.1 to 0.3. This is mainly attributed to an increment in the EDL thickness and induced potential (see Fig. 4(a)) with an increment in the PEL thickness from 0.1 to 0.3. An increment in *d* (from 0.1 to 0.3) increases the fluid velocity (see Fig. 5(b)) and culminates in an enhancement in the flow rate in the soft nanochannel as observed in Fig. 9(a). Next, in Fig. 9(b), we observe an increasing nature in the flow rate enhancement with a decrement in the drag parameter *α* from 5 to 0.1 even for the all the values of *n* considered, mainly attributed to the reduction in the friction drag offered by the PEL to the fluid with a decrement in *α*. Since a decrement in *α* weakens the friction drag applied by the PEL on the fluid, the fluid velocity as well as the flow rate in the channel enhances with a decrement in *α* as observed in Fig. 9(b). However, in Fig. 9(b), we observe two different trends of the variation of *Q*_*SC*_/*Q*_*RC*_ with a change in power-law index *n* from 0.7 to 1.5 for *α* = 5. The flow rate ratio *Q*_*SC*_/*Q*_*RC*_ initially increases with an increment in *n* from 0.7 to 1, which is then followed by a decreasing trend as *n* increases further from 1.0 to 1.5. We here take an effort to figure out the physics behind this observation as follows: it may be mentioned here that the higher friction drag on the flow field originating from the higher value of *α*(=5) leads to a relatively higher reduction in flow velocity for shear-thinning fluids (*n* < 1), since the apparent viscosity is lesser for this class of fluids. As such, the relative reduction in flow velocity and so is the flow rate becomes relatively lesser for the underlying transport of shear-thinning fluids through a soft channel, attributed to the stronger electrical forces stemming from the ionic interaction between electrolyte layer and PEL. Notably, a relatively lesser reduction in flow rate through soft nanochannel (*Q*_*SC*_) than the rigid channel (*Q*_*RC*_) leads to an increasing trend of *Q*_*SC*_/*Q*_*RC*_ in the shear-thinning regime (*n* < 1) as witnessed in Fig. 9(b). On the other hand, we observe a decreasing trend of *Q*_*SC*_/*Q*_*RC*_ in the shear-thickening regime with increasing value of *n* i.e., with increasing non-Newtonian behavior of the fluid, to be precise, with the increasing value of apparent viscosity of the fluid. The decrement in the flow velocity and its effect on the flow rate through the soft nanochannel is relatively higher than the rigid nanochannel, leading to a decreasing trend of *Q*_*SC*_/*Q*_*RC*_ in the shear-thickening regime that too observed in Fig. 9(b). We attribute this observation to the effect of rheology modulated enhancement in viscous drag on the flow dynamics. We would like to convey the following message in the context of the variation of flow rate ratio (*Q*_*SC*_/*Q*_*RC*_) vs. *n*, obtained for a relatively higher value drag parameter *α*(=5) as follows: in the shear-thinning regime (*n* < 1), the electroosmotic body force originating from the EDL phenomenon due to a relatively stronger ionic interaction between PEL and electrolyte layer takes a dominating role on the underlying transport, while the rheology modulated viscous resistance governs the flow dynamics in the shear-thickening regime.

## Concluding Remarks

In summary, we discuss the electrohydrodynamics of non-Newtonian fluids in a polyelectrolyte grafted soft narrow fluidic channel with the consideration of electrical double layer originating from the ionic interactions between PEL and electrolyte layer. We consider the Ostwald-de-Waele power-law model for describing the non-Newtonian fluid rheology in this analysis. The results unveil that the variation in the charge concentration in PEL as well as in the electrolyte layer, thickness of PEL and polymeric density affect the electroosmotic body force being applied on the fluid mass in the channel, which upon interacting with the viscous force as modulated by the fluid rheology, alters the velocity distribution and flow rate variation in the channel non-intuitively. We investigate through this analysis that, a higher PEL thickness, lesser Darcy drag and adequately low electrolyte concentration leads to a high flow rate in the channel for shear-thinning fluids, while the flow rate of shear-thickening fluids gets reduced for lower PEL thickness, higher Darcy drag and higher electrolyte concentration. Also, we show a relative enhancement in the net throughput through the soft narrow fluidic channel as compared to that of the rigid channel for different parameters and identify two distinct regimes. It is shown that a decrement in PEL thickness and increment in drag parameter leads to a reduction over the relative increment in the net throughput through the channel. These observations are suggestive off having polyelectrolyte layer with low grafting density and higher charge concentration of PEL essentially for the enhancement of net throughput in the channel. A continual decrement in the relative enhancement of the net throughput or volumetric flow rate through the soft nanochannel with increasing *n* (i.e. with increasing non-Newtonian behavior of fluid) for all *d* and *α* signifies the pronounced effect of the viscous resistance to the flow field, attributed primarily to the increment in apparent viscosity with *n*.
